# Community nurses’ willingness to implement Advance Care Planning (ACP) and their attitudes toward death: current status and correlation analysis

**DOI:** 10.3389/fmed.2025.1573314

**Published:** 2025-06-06

**Authors:** Qiyan Peng, Yujie Dong, Qin Dai, Chi Zhang, Xiaoling Li

**Affiliations:** ^1^Leshan Vocational and Technical College, Leshan, China; ^2^West China Hospital, Sichuan University, Chengdu, Sichuan, China

**Keywords:** community nurses, Advance Care Planning, death attitudes, cross-sectional survey, correlation analysis

## Abstract

**Objective:**

This study aims to evaluate the current status of community nurses’ willingness to implement Advance Care Planning (ACP) and their attitudes toward death, and to explore the correlation between these variables, providing a basis for improving ACP implementation strategies.

**Methods:**

A convenience sampling method was employed to select 317 nurses from 15 community health service centers in Chengdu. Data were collected using a general information questionnaire, the ACP Implementation Willingness Scale, and the Death Attitude Profile-Revised (DAP-R).

**Results:**

The overall score for community nurses’ willingness to implement ACP was 56.87 ± 9.73, indicating a moderately high level of willingness. In terms of death attitudes, the highest score was observed in the “natural acceptance” dimension (3.76 ± 0.63), followed by “death avoidance” (3.20 ± 0.81) and “death fear” (3.07 ± 0.80). A significant negative correlation was found between ACP implementation willingness and both “death fear” and “death avoidance” (*r* = −0.358 to −0.414, *P* < 0.001). In contrast, a positive correlation was observed between ACP implementation willingness and “natural acceptance,” “approach acceptance,” and “escape acceptance” (*r* = 0.151 to 0.494, *P* < 0.001). Multiple stepwise linear regression analysis revealed that age, education level, previous exposure to death education training, familiarity with or exposure to ACP, and death attitudes (particularly “death avoidance” and “natural acceptance”) were the main factors influencing nurses’ willingness to implement ACP, explaining 40.4% of the total variance.

**Conclusion:**

Community nurses’ willingness to implement ACP is moderately high; however, their understanding of ACP remains limited. Death attitudes are primarily characterized by “natural acceptance,” though negative attitudes toward death, such as “death avoidance,” persist. Enhancing death education and training, improving nurses’ understanding of ACP, and fostering a more accurate perception of death are essential to facilitate broader ACP implementation in the general population.

## 1 Introduction

As of 2022, according to the United Nations Population Division, the global population aged 65 and above has reached 727 million, and it is projected to more than double to 1.5 billion by 2050, representing 16% of the world’s total population (1). This demographic shift underscores the accelerating trend of societal aging, with similar patterns observed across developed and developing nations. Along with this global aging trend, the incidence of chronic diseases such as cancer, cardiovascular disease, and diabetes has risen sharply, exacerbating the mortality burden and increasing the need for end-of-life care. Death has thus become an unavoidable and pressing topic in healthcare, particularly in the context of aging populations worldwide.

Advance Care Planning (ACP) is an essential process through which individuals, while still mentally competent, make autonomous decisions regarding their future healthcare, based on their values and life experiences (2). ACP enables individuals to express their preferences for end-of-life care and to engage in discussions with family members and healthcare providers to ensure that their wishes are honored when they are no longer able to communicate them. ACP has become a cornerstone of palliative care in countries such as the United States, the United Kingdom, Canada, and Australia, where it is integrated into healthcare policies and is widely endorsed by international healthcare organizations such as the World Health Organization (WHO) (3). These countries have recognized the importance of ACP in enhancing the quality of life for patients and ensuring that care is aligned with patients’ values.

In the context of global health systems, primary healthcare institutions have been identified as optimal settings for the promotion and implementation of ACP. The World Health Organization (WHO) and the European Commission emphasize the need for community-based healthcare interventions that empower individuals to make informed decisions about their health, particularly in the face of chronic and terminal illnesses (4). Community healthcare settings, as essential parts of primary healthcare systems, play a crucial role in the dissemination of health policies and the facilitation of public communication (5). Community nurses, as key members of the primary care team, are critical in the successful implementation of ACP, supporting patients and families through discussions and ensuring that the care provided reflects the patients’ values and preferences. While ACP has been widely recognized as a fundamental component of palliative care in high-income countries, its implementation remains in the developmental and exploratory stages in many other regions. In recent years, international organizations have emphasized the need to incorporate ACP into primary healthcare systems, especially in response to the growing number of elderly individuals worldwide. However, the research on ACP in community healthcare settings, particularly in low- and middle-income countries, remains limited (6).

This study aims to investigate the current status of community nurses’ willingness to implement ACP and to analyze the factors influencing this willingness. The goal is to inform the development of targeted intervention measures for ACP training tailored to community nurses, thereby contributing to the broader implementation of ACP in community healthcare systems globally. The findings of this study will provide a valuable foundation for further research and the development of evidence-based strategies for integrating ACP into community healthcare settings.

Research questions:

What is the current status of community nurses’ willingness to implement ACP and their attitudes toward death?

How do demographic factors and death attitudes correlate with ACP implementation willingness?

Hypotheses:

Nurses with higher education levels exhibit stronger willingness to implement ACP.

Negative death attitudes (e.g., death avoidance) are inversely correlated with ACP willingness.

## 2 Materials and methods

### 2.1 Study participants

A convenience sampling method was employed to select nurses from 15 community health service centers in the main urban districts of Chengdu between June and July 2024. Nurses who met the inclusion criteria were invited to participate in the study.

Inclusion criteria:

(1) Full-time nurses currently employed at the community health service centers;
(2) Voluntary participation and informed consent to the study.

Exclusion criteria:

(1) Interns or nurses on advanced training programs;
(2) Nurses on long-term sick leave or leave of absence.

### 2.2 Sample size estimation

This study adopts a cross-sectional design. According to the principle that the sample size should be at least 5 to 10 times the number of independent variables (7), the study includes 14 items of general demographic data, 5 dimensions of the second scale, and 3 dimensions of the third scale. Considering a 20% invalid response rate, the estimated sample size ranges from 121 to 242 participants.

### 2.3 Methods

#### 2.3.1 Survey instruments

(1) General Demographic Questionnaire

The General Demographic Questionnaire included variables (e.g., gender, age, marital status, educational background, professional title, years of work experience, and religious beliefs) consistently identified as key predictors of ACP engagement in prior nursing research. Their selection aligns with recommendations from standardized instruments for assessing healthcare professionals’ roles in end-of-life care planning.

(2) Death Attitude Profile-Revised (DAP-R)

The DAP-R was originally developed by Fry and Wong (8) in 1994 and later adapted for use in Chinese mainland and Taiwan by Wang et al. (9) following cross-cultural adaptation principles. The scale consists of five dimensions, with a total of 32 items, covering fear of death (items 1/2/7/18/20/21/32): Negative emotional reactions to death-related events (e.g., “I feel afraid when I think about death”), death avoidance (items 3/10/12/19/26): Reducing anxiety by avoiding death-related topics or behaviors (e.g., “I try to avoid talking about death”), natural acceptance (items 6/14/17/24/30): Viewing death as a natural part of life (e.g., “Death is an integral part of life”), approach acceptance (items 4/8/13/15/16/22/25/27/28/31): Accepting death due to religious beliefs or hope for an afterlife (e.g., “I believe death leads to a better place”), and escape acceptance (items 5/9/11/23/29): Accepting death as relief from unbearable suffering (e.g., “Death is a way to escape when life becomes intolerable”). Fear of death and death avoidance are considered negative attitudes, while natural acceptance, approach acceptance, and escape acceptance represent positive attitudes. The Likert 5-point scale was used, with scores ranging from 1 (strongly disagree) to 5 (strongly agree). In this study, the total score was not calculated; instead, the mean score for each dimension was derived by dividing the total score for that dimension by the number of items. Higher scores indicate a stronger alignment with that particular attitude toward death. The Cronbach’s alpha coefficient of this scale is 0.88, indicating excellent internal consistency (10).

(3) Willingness to implement Advance Care Planning (ACP) scale for healthcare workers

This scale, developed by Shi et al. (11), is used to assess healthcare workers’ willingness to implement ACP. The scale consists of three dimensions—cognitive, attitudinal, and behavioral intention—with a total of 15 items. Responses were rated on a 5-point Likert scale ranging from 1 (strongly disagree) to 5 (strongly agree), with a total score range of 15 to 75. Higher scores indicate stronger willingness to implement ACP. Pre-testing showed that the Cronbach’s alpha for the entire scale was 0.966, with Cronbach’s alpha values for individual dimensions ranging from 0.874 to 0.934.

### 2.4 Data collection

Prior to data collection, support was obtained from the nursing departments of the surveyed community health service centers, and communication and coordination were carried out. The survey was conducted using an online questionnaire platform (Wenjuanxing). If participants had any questions about the questionnaire, researchers provided consistent clarification. To avoid duplicate submissions, the electronic survey was restricted so that each WeChat ID could only submit one response. The survey had set minimum and maximum completion times—responses completed in less than 5 min or longer than 60 min were considered invalid. A total of 340 questionnaires were distributed, and 23 invalid questionnaires (those with identical responses for all items or completion times outside the acceptable range) were excluded. Finally, 317 valid questionnaires were collected, yielding an effective response rate of 93.2%.

### 2.5 Statistical analysis

Data collected through Wenjuanxing were exported and cross-checked by two researchers before statistical analysis. Descriptive statistics for continuous variables were presented as means and standard deviations (X̄ ± S), while categorical variables were presented as frequencies and percentages. Data normality was assessed using the Shapiro–Wilk test; variables violating normality assumptions were analyzed with non-parametric tests. Differences in ACP implementation willingness scores across different demographic characteristics were analyzed using *t*-tests and analysis of variance (ANOVA). Pearson correlation analysis was used to examine the relationship between death attitudes and ACP implementation willingness. Multivariable analysis was conducted using multiple linear regression. Statistical significance was set at *P* < 0.05.

## 3 Results

### 3.1 Demographic characteristics of community nurses

A total of 340 questionnaires were distributed, of which 317 were successfully returned. Among the 317 community nurses surveyed, 307 (96.8%) were female and 10 (3.2%) were male, with an average age of (30.77 ± 7.76) years. Detailed demographic information is presented in Table 1.

**TABLE 1:** Comparison of ACP implementation willingness scores among community nurses with different demographic characteristics (*n* = 317).

Item	Number (percentage %)	Score	F/t value	*P*-value
**Gender**			2.390	**0.035**
Male	10 (3.2)	3.55 ± 0.31		
Female	307 (96.8)	3.80 ± 0.66		
**Age**			3.108	**0.027**
≤ 30	176 (55.5)	3.71 ± 0.65		
31–40	110 (34.7)	3.85 ± 0.65		
41–49	20 (6.3)	4.11 ± 0.52		
≥ 50	11 (3.5)	3.92 ± 0.60		
**Marital status**			6.143	**0.002**
Unmarried	128	3.65 ± 0.61		
Married	183	3.90 ± 0.66		
Divorced	6	3.52 ± 0.47		
Widowed	–	–		
**Education level**			3.569	**0.029**
Master’s degree or above	4	4.47 ± 0.94		
Bachelor’s degree	191	3.83 ± 0.63		
Associate degree or below	122	3.71 ± 0.66		
**Title**			2.143	0.095
Associate chief nurse or chief nurse	14	3.98 ± 0.59		
Supervisor nurse	102	3.88 ± 0.62		
Nurse	111	3.78 ± 0.69		
Staff nurse	90	3.67 ± 0.62		
**Years of work experience**			5.901	**0.003**
< 5	115	3.63 ± 0.65		
5–10	72	3.86 ± 0.62		
> 10	130	3.90 ± 0.64		
**Religious belief**			0.132	0.895
Yes	23	3.77 ± 0.61		
No	294	3.79 ± 0.65		
**Hospitalization experience**			1.404	0.161
Yes	199	3.83 ± 0.66		
No	118	3.72 ± 0.62		
**Grief experience**			0.431	0.667
Yes	187	3.80 ± 0.65		
No	130	3.77 ± 0.65		
**Funeral participation (e.g., ceremony, burial)**			0.173	0.863
Yes	286	3.79 ± 0.62		
No	31	3.77 ± 0.85		
Contact with terminally ill patients			0.481	0.631
Yes	265	3.78 ± 0.66		
No	52	3.83 ± 0.60		
**Family discussion of death-related topics**			4.400	**0.005**
Never discuss	68	3.71 ± 0.69		
Occasionally discuss	194	3.74 ± 0.63		
Discuss when necessary, but the atmosphere is unnatural	37	3.97 ± 0.64		
Frequently discuss openly	18	4.22 ± 0.47		
**Participation in death education training**			4.769	< **0.001**
Yes	60	4.14 ± 0.60		
No	257	3.71 ± 0.63		
**Familiarity with or exposure to ACP**			4.380	< **0.001**
Yes	63 (19.9)	4.10 ± 0.60		
No	254 (80.1)	3.71 ± 0.64		

Bold *p*-values indicate statistical significance (*p* < 0.05). F/t value: F-value: Derived from one-way ANOVA, used for comparisons among ≥ 3 groups (e.g., age, marital status). t-value: Derived from independent samples *t*-test, used for comparisons between two groups (e.g., gender, religious belief).

### 3.2 ACP implementation willingness and death attitudes scores of community nurses

The overall score for community nurses’ willingness to implement ACP was (56.87 ± 9.73). The scores for each dimension and the mean scores for the individual items are presented in Table 2. In terms of death attitudes, the mean scores for the dimensions, ranked from highest to lowest, were as follows: natural acceptance, death avoidance, death fear, escape acceptance, and approach acceptance. Detailed scores are provided in Table 3.

**TABLE 2:** ACP implementation willingness and scores of each dimension in community nurses (*n* = 317).

Dimension	Number of items	Score range	Dimension score	Mean score per item
Cognitive	6	6–30	22.19 ± 4.61	3.69 ± 0.77
Attitude	4	4–20	15.74 ± 2.78	3.94 ± 0.69
Behavioral intention	5	5–25	18.94 ± 3.60	3.79 ± 0.72
Total score	15	15–75	56.87 ± 9.73	3.79 ± 0.65

**TABLE 3:** Scores of each dimension of death attitudes in community nurses (*n* = 317).

Dimension	Number of items	Score range	Dimension score	Item mean score
Death anxiety	7	7–35	21.02 ± 5.29	3.07 ± 0.80
Death avoidance	5	5–25	15.51 ± 4.11	3.20 ± 0.81
Natural acceptance	5	5–25	18.80 ± 3.14	3.76 ± 0.63
Approach acceptance	10	10–50	28.25 ± 8.21	2.82 ± 0.82
Escape acceptance	5	5–25	14.47 ± 4.52	2.89 ± 0.90

### 3.3 Univariate analysis of community nurses’ ACP implementation willingness scores

As shown in Table 1, significant differences in ACP implementation willingness scores were observed among community nurses based on gender, age, marital status, education level, years of work experience, frequency of family discussions about death-related topics, participation in death education training, and familiarity with or exposure to ACP (*P* < 0.05).

### 3.4 Correlation analysis between death attitudes and ACP implementation willingness in community nurses

Pearson correlation analysis revealed that the total score for community nurses’ willingness to implement ACP was negatively correlated with death fear and death avoidance, and positively correlated with natural acceptance, approach acceptance, and escape acceptance. The detailed results are presented in Table 4.

**TABLE 4:** Pearson correlation coefficients between ACP willingness and death attitudes (*r*).

Variable	Death fear	Death avoidance	Natural acceptance	Approach acceptance	Escape acceptance
ACP implementation willingness total score	−0.358	−0.414	0.494	0.169	0.151
*P*-value	< 0.001	< 0.001	< 0.001	0.003	0.007

### 3.5 Multivariable stepwise linear regression analysis of factors influencing community nurses’ ACP implementation willingness

A multivariable stepwise linear regression analysis was conducted with the total score for community nurses’ willingness to implement ACP as the dependent variable, and variables showing statistical significance in univariate analysis and correlation analysis as independent variables (the coding of these variables is shown in Table 5). The results indicated that the key factors influencing ACP implementation willingness were age, education level, participation in death education training, familiarity with or exposure to ACP, death avoidance, and natural acceptance (*P* < 0.05), which together explained 40.4% of the total variance. Detailed results are provided in Table 6.

**TABLE 5:** Coding method for independent variables.

Independent variable	Coding
Age	≤ 30 = 1; 31–40 = 2; 41–49 = 3; ≥ 50 = 4
Education level	Associate degree or below = 1; bachelor’s degree = 2; master’s degree or above = 3
Death education	No = 0; yes = 1
Familiarity with or exposure to ACP	No = 0; yes = 1
Death avoidance	Raw values input
Natural acceptance	Raw values input

**TABLE 6:** Stepwise linear regression of factors influencing ACP implementation willingness (*n* = 317).

Model	Unstandardized coefficients		Standardized coefficients	95% confidence interval (CIs)	*t*	Significance
	**B**	**Standard error**	**Beta**			
Constant	3.839	0.319	–	(3.213, 4.465)	12.034	< 0.001
Age	0.089	0.039	0.105	(0.013, 0.165)	2.297	0.022
Education level	0.130	0.058	0.102	(0.016, 0.244)	2.250	0.025
Death education	0.211	0.086	0.128	(0.042, 0.380)	2.438	0.015
Familiarity with or exposure to ACP	0.176	0.085	0.108	(0.009, 0.343)	2.073	0.039
Death avoidance	−0.243	0.035	−0.309	(−0.312, −0.174)	−6.934	< 0.001
Natural acceptance	0.418	0.046	0.405	(0.328, 0.508)	9.067	< 0.001

All *p*-values are two-tailed.

## 4 Discussion

### 4.1 Current status of community nurses’ ACP implementation willingness

Advance Care Planning (ACP) is critical in facilitating patient-centered care, particularly for individuals facing end-of-life decisions. ACP empowers patients to communicate their treatment preferences and ensures that healthcare professionals respect these preferences, which significantly enhances quality of life and supports autonomy (12, 13). This study highlights that the overall willingness of community nurses to implement ACP is moderate (56.87 ± 9.73), with the highest scores in attitude and behavioral intention. However, nurses’ cognitive understanding of ACP remains an area for improvement, as suggested by the relatively lower score in the cognition dimension. This finding is consistent with global trends where healthcare providers, including nurses, often lack adequate training in ACP, which hinders its effective implementation (14, 15). In China, ACP implementation is not yet mandated as a routine nursing responsibility. Institutional policies vary, and nurses’ engagement in ACP often depends on individual willingness and organizational support.

The moderate willingness observed in this study may reflect cultural, institutional, and educational barriers to ACP adoption. In China, the slow development of ACP practices, coupled with a lack of formal education in nursing curricula, limits community nurses’ comprehensive understanding of ACP (16). Although many nurses understand the importance of respecting patient autonomy and alleviating suffering at the end of life, the absence of clear institutional policies and guidelines on ACP implementation in China further complicates its integration into routine nursing practice (17). A critical issue that needs to be addressed is the nurses’ lack of exposure to ACP discussions, as fewer than 20% of surveyed nurses reported prior knowledge or exposure to ACP. Studies from other countries report similar gaps, where ACP remains underutilized despite its potential to improve patient outcomes (18). The findings in this study underscore the need for structured training programs on ACP, which would not only enhance knowledge but also provide nurses with the necessary tools to facilitate ACP conversations effectively.

### 4.2 Community nurses’ attitudes toward death

Community nurses’ attitudes toward death play a significant role in their ability to engage in end-of-life discussions, including ACP. The results of this study reveal that community nurses scored highest on “natural acceptance” (3.76 ± 0.63), indicating that most nurses view death as a natural part of life. This aligns with findings from studies that suggest healthcare workers, particularly those with more exposure to end-of-life care, tend to develop more accepting attitudes toward death (19–21). Nurses’ higher acceptance of death could facilitate more open discussions about ACP with patients and families.

However, the scores on “death anxiety” (3.07 ± 0.80) and “death avoidance” (3.20 ± 0.81) were also relatively high, reflecting lingering discomfort and fear regarding death. This finding suggests that while nurses may intellectually accept death, they may not be emotionally prepared to address death-related topics. This contradiction highlights a critical challenge in ACP implementation, as emotional discomfort and fear of death can interfere with the effective communication of ACP to patients and families. Similar findings were reported by Draper et al. (22), who noted that healthcare providers with high levels of death anxiety may avoid discussing end-of-life issues, even when they recognize their importance.

### 4.3 Factors influencing community nurses’ ACP implementation willingness

#### 4.3.1 The role of age in ACP implementation willingness

This study found that older community nurses exhibited stronger willingness to implement ACP. Aging is associated with increased exposure to death and terminal care, leading to a more profound understanding of the necessity and benefits of ACP. Older nurses, with their accumulated clinical experience, tend to have more realistic expectations about death and the importance of ensuring that patients’ final wishes are respected (14). This finding underscores the value of experiential learning in nursing practice, where older nurses may be more inclined to engage in sensitive topics like ACP due to their broader understanding of the patient care continuum.

#### 4.3.2 Higher education levels correlate with increased ACP willingness

Nurses with higher education levels demonstrated stronger ACP implementation willingness. This correlation can be attributed to several factors, including enhanced knowledge, critical thinking skills, and professional confidence. Higher education in nursing often includes comprehensive training in palliative care, ethics, and end-of-life care, all of which are essential components of ACP. Nurses with advanced education are also more likely to advocate for patient autonomy and integrate ACP into routine care practices (23). Moreover, the development of critical thinking skills enables nurses to address complex patient concerns and articulate the benefits of ACP more effectively.

#### 4.3.3 Impact of death education on ACP implementation willingness

Death education has been shown to improve nurses’ understanding of death-related issues and foster a more rational, compassionate approach to end-of-life care (24). Nurses with death education training in this study displayed a stronger willingness to engage in ACP discussions. This aligns with previous studies that suggest death education helps nurses overcome death-related anxiety, increases their competence in managing end-of-life discussions, and improves their confidence in implementing ACP (25). Incorporating death education into nursing curricula and professional development programs could significantly enhance nurses’ preparedness to engage in these critical conversations.

#### 4.3.4 The influence of death attitudes on ACP implementation willingness

##### 4.3.4.1 Negative impact of death avoidance

As noted in the correlation analysis, death avoidance was negatively correlated with ACP implementation willingness. Nurses who avoid confronting death-related topics are less likely to recognize the importance of ACP, leading to reluctance in engaging with patients and families about their end-of-life preferences. This avoidance may be rooted in cultural factors, personal fears, or a lack of emotional preparation. Addressing these attitudes through targeted training and support programs is crucial for increasing nurses’ confidence in discussing ACP and end-of-life issues (26).

##### 4.3.4.2 Positive impact of natural acceptance

Conversely, natural acceptance of death positively influenced ACP implementation willingness. Nurses who accept death as a natural process are more likely to appreciate the role of ACP in respecting patients’ wishes and providing dignified care. This finding is consistent with other research indicating that positive death attitudes are associated with greater involvement in palliative and end-of-life care (27). Nurses with a natural acceptance of death are more likely to initiate and participate in ACP discussions, which is essential for ensuring that patients’ healthcare decisions align with their values and preferences. Li et al. (28) also found that nurses with positive attitudes toward death were more likely to participate in and promote end-of-life care, including ACP.

## 5 Conclusion

This study highlights several key factors influencing the willingness of community nurses to implement ACP, including age, education level, exposure to death education, and death attitudes. The results emphasize the need for comprehensive training programs that address both the technical aspects of ACP and the emotional challenges related to death. Providing community nurses with the tools to engage in end-of-life care discussions not only improves their competence but also enhances the quality of care provided to patients at the end of life. Given the increasing importance of ACP in patient-centered care, healthcare systems must invest in educating and supporting nurses to ensure that ACP is effectively implemented, aligning with both professional standards and patient needs.

### 5.1 Limitations and future directions

This study has several limitations. The cross-sectional design and reliance on a single geographic region (Chengdu) restrict causal inferences and generalizability, as cultural and socioeconomic disparities in other areas (e.g., rural vs. urban, eastern vs. western China) may shape nurses’ attitudes toward death and ACP willingness. Additionally, the use of convenience sampling and online questionnaires may introduce selection bias and social desirability bias in self-reported data. Future research should adopt stratified sampling across diverse regions, integrate mixed-methods approaches (e.g., qualitative interviews) to validate findings, and employ longitudinal designs to assess ACP willingness dynamics over time. Expanding investigations to include patients’ and families’ perspectives would further clarify barriers and facilitators of ACP implementation in community settings.

## Data Availability

The original contributions presented in this study are included in this article/supplementary material, further inquiries can be directed to the corresponding author.
